# The opposite effects of nandrolone decanoate and exercise on anxiety levels in rats may involve alterations in hippocampal parvalbumin–positive interneurons

**DOI:** 10.1371/journal.pone.0189595

**Published:** 2017-12-12

**Authors:** Dragica Selakovic, Jovana Joksimovic, Ivan Zaletel, Nela Puskas, Milovan Matovic, Gvozden Rosic

**Affiliations:** 1 Department of Physiology, Faculty of Medical Sciences, University of Kragujevac, Kragujevac, Serbia; 2 Institute of Histology and Embryology “Aleksandar Đ. Kostić”, School of Medicine, University of Belgrade, Belgrade, Serbia; 3 Deparment of Nuclear Medicine, Faculty of Medical Sciences University of Kragujevac, Clinical Centre "Kragujevac", Kragujevac, Serbia; University of Queensland, AUSTRALIA

## Abstract

The aim of this study was to evaluate the behavioral effects of chronic (six weeks) nandrolone decanoate (ND, 20 mg/kg, s.c., weekly in single dose) administration (in order to mimic heavy human abuse), and exercise (swimming protocol of 60 minutes a day, five days in a row/two days break), applied alone and simultaneously with ND, in male rats (n = 40). Also, we evaluated the effects of those protocols on hippocampal parvalbumin (PV) content and the possible connection between the alterations in certain parts of hippocampal GABAergic system and behavioral patterns. Both ND and exercise protocols induced increase in testosterone, dihydrotestosterone and estradiol blood levels. Our results confirmed anxiogenic effects of ND observed in open field (OF) test (decrease in the locomotor activity, as well as in frequency and cumulative duration in the centre zone) and in elevated plus maze (EPM) test (decrease in frequency and cumulative duration in open arms, and total exploratory activity), that were accompanied with a mild decrease in the number of PV interneurons in hippocampus. Chronic exercise protocol induced significant increase in hippocampal PV neurons (dentate gyrus and CA1 region), followed by anxiolytic-like behavioral changes, observed in both OF and EPM (increase in all estimated parameters), and in evoked beam-walking test (increase in time to cross the beam), compared to ND treated animals. The applied dose of ND was sufficient to attenuate beneficial effects of exercise in rats by means of decreased exercise-induced anxiolytic effect, as well as to reverse exercise-induced augmentation in number of PV immunoreactive neurons in hippocampus. Our results implicate the possibility that alterations in hippocampal PV interneurons (i.e. GABAergic system) may be involved in modulation of anxiety level induced by ND abuse and/or extended exercise protocols.

## Introduction

Anabolic androgenic steroids (AASs) comprise a large class of synthetic compounds made up of testosterone and its derivatives. AASs have an important role in the treatment of various chronic diseases [1]. Top athletes use AASs in order to improve physical performance [2]. In the last few decades, the abuse of AASs has been widely spread among the adolescent males [3], and became a problem even among non-athletes, representing a public-health concern. Increased prevalence of behavioral disorders, including unprovoked aggression and violence, has been reported in AASs abusers [4]. Long-term AASs abusers are characterized by high level of anxiety and extreme mood-swings [5].

Studies performed on animals also reported AASs modulation of anxiety behavior. Results obtained from animal experiments are controversial. Some authors reported anxiolytic-like effects [6], while other studies showed anxiogenic effects of AASs in rats [7]. However, it should be emphasized that some of those studies were performed on different species, with different classes, protocols and doses of AASs.

Beneficial effects of exercise on physical performance are well known. Reports for the impact of exercise on cognitive and emotional aspects of behavior are much more recent [8]. The behavioral effects of exercise depend on various features, such as training length (acute vs. chronic), modality and control of the exercise (e.g., voluntary wheel running vs. forced treadmill training or swimming), intensity of the exercise (self-selected vs. manipulated), and duration of the exercise [9]. It has been shown that certain types of exercise protocols (mild or moderate exercise) have anxiolytic and antidepressant effects that influence the management of stress [10], while anxiogenic outcome was observed following high intensity exercise [11]. Also, chronic physical activity induced behavioral changes in animals [12], such as anxiolytic effects in rats [13] and anxiogenic effects in mice [14], depending on the type of exercise protocol.

Simultaneous application of AASs and chronic exercise showed contradictory results, possibly due to different protocols both for exercise and AASs administration. However, the results of numerous studies confirmed the attenuation of beneficial effects of exercise after AAS administration in rats [15].

The hippocampus is a structure that has a key role in cognitive and emotional processes. Hippocampal formation has two main groups of neurons: principal neurons, responsible for extrahippocampal connections, and interneurons (predominantly GABAergic), responsible for local connections within the hippocampus [16]. γ-Aminobutyric acid (GABA) is a major inhibitory neurotransmitter in the mammalian brain. GABA interneurons are widely distributed in several regions of brain and have a major role in modulating local noradrenergic, dopaminergic, serotonergic and glutamatergic neuronal circuitry. GABAergic dysfunction has been reported to be associated with depressive symptoms [17], mood disorders [18], bipolar disorder [19] and post-traumatic stress disorder [20]. Hippocampal GABAergic neurons, according to specific immunoreactivity, are divided into subpopulations: neuropeptide Y-, somatostatin-, dynorphin- and parvalbumin-positive interneurons. Parvalbumin (PV) belongs to the group of calcium-binding proteins and it is specific for vertebrates [21]. PV-positive neurons are widely distributed cell population that is present in different parts of the central nervous system, with a respectable number in hippocampus [22].

Since hippocampus plays one of the key roles in mood modulation and may be involved in control of some specific behavioral patterns [23], alterations in hippocampal PV content have been proposed as a possible explanation for exercise-induced behavioral changes. It has been reported that behavioral alterations induced by various types of exercise protocols were accompanied with specific modification in hippocampal parvalbumin expression [24–26]. Although, there is no literature data concerning the alterations in hippocampal PV interneurons following AASs administration and its connection to behavioral alterations, the results that showed testosterone propionate-induced changes in spine density on neurons in the limbic system, including hippocampus, and their excitability [27], made good connection to the possible influence of AASs on hippocampal GABAergic system. Since it has been reported that both various exercise protocols and AASs administration (in different doses) induced significant changes in sex hormone levels, and therefore can affect the neurogenesis in various brain regions, including hippocampus [28], it seems relevant to evaluate the specific effects of altered sex hormone levels on hippocampal PV content and its possible behavioral manifestations, as a start point for investigation of numerous AASs actions in generating of mood alterations that should be followed by more extensive research, as presented by Troakes and Ingram [29].

Considering the fact that nandrolone decanoate (ND) is one of the most commonly used AAS, the aim of this study was to evaluate the effects of chronic ND administration and exercise (swimming protocol) on behavioral changes in rats by means of specific behavioral tests, as well as on hippocampal PV content. Serum testosterone (T), dihydrotestosterone (DHT) and estradiol (E2) were determined in order to quantify the effects of chronic AAS treatment and exercise protocols by means of the impact on sex hormone levels in blood. Additionally, we planned to estimate the possible connection between the alterations in certain parts of hippocampal GABAergic system and behavioral patterns following chronic ND abuse and exercise protocols.

## Materials and methods

### Ethic statement

This study, including pretreatment procedures, was carried out in strict accordance with the European Directive for welfare of laboratory animals N° 86/609/EEC and principles of Good Laboratory Practice (GLP). The protocol was approved by the by Ethical Committee of the Faculty of Medical Sciences, University of Kragujevac, Serbia.

### Animals and treatment

Due to fact that there is consensus about more common AAS abuse among males than females [30–32], we performed this study on male Wistar albino rats (three-month-old, 350–400 g, n = 40). Animals were housed in groups of 3–5 per cage, in an environment maintained at 23±1°C, with a 12/12h light/dark cycle. The animals had free access to food and water. The experimental groups were as follows: control group (C group, n = 8), nandrolone decanoate group (ND group, n = 12), exercise group (E group, n = 11) and nandrolone decanoate plus exercise group (ND+E group, n = 9). Nandrolone decanoate (DEKA 300, SteroxLab, EU), in a final dose of 20 mg/kg, was administered subcutaneously (s.c.) once weekly for 6 weeks. The supraphysiological dose of ND was used in order to mimic the doses for heavy human AAS abusers [33, 34]. Exercise group performed swimming training protocol for 6 weeks (60 minutes a day, five days in a row, two days break) in a heated (32±1°C) glass swimming pool (60x75x100 cm) in a group of 3–5 animals. The exercise protocol was performed following the adaptive period (20 minutes of swimming per day for one week) in order to reduce water-induced stress [35]. The duration of the swimming trial was defined on the basis of a previous report as the protocol sufficient to induce immunohistochemical alterations in certain brain regions, such as hippocampus and prefrontal cortex that are reported to be involved in behavior alterationsin rats [36]. Since the swimming is an inherited behavior pattern among rodents [37], this protocol was used as an exercise model of endurance training. ND+E group had received 20 mg/kg of ND (s.c.) weekly and performed the same swimming protocol as exercise group for six weeks. Control and exercise groups received approximately the same amount of sterilized olive oil in the same manner (by means of volume and route of administration) as ND and ND+E groups received therapy. In order to eliminate the difference between exercise and non-exercise groups caused by water immersion, rats from sedentary (control and ND) groups were placed in the same water tank (7 cm water depth) for short time (2 minutes) each day of the training protocol, also in groups of 3–5 animals in order to maintain the same social context of swimming training. The experimenter was present during the whole swimming protocol monitoring the rats. After the swimming session rats were towel dried and placed in a clean cage.

Two days after the protocols were finished (to maintain the design established in this investigation—5 days of swimming / 2 days break), the rats were placed in a testing room for 1–2 h to accommodate before behavioral testing (approximately at 7 a.m.). The same-housed animals were tested on the same day, starting at approximately 9 a.m. All tests were performed under proper conditions of silence and illumination for this kind of behavioral testing (the room illuminated with controlled light, ~100 lx) as previously described [38]. All three behavioral tests were performed one by one (for all investigated groups) in a following order: open field (OF) test, elevated plus maze (EPM) test, and evoked beam-walking (EBW) test. Inter-trial-interval of approximately 15 minutes between the two consecutive tests was allowed in order to avoid (minimize) the cumulative effects of the repeated anxiety-provoking testing. During the behavioral testing, mazes were cleaned following the trial for each animal to remove possible interfering scents. After the completion of behavioral tasks (approximately at 1 p.m.) rats were decapitated following short-term narcosis induced by intraperitoneal application of ketamine (10 mg/kg) and xylazine (5 mg/kg), trunk blood samples were collected for determination of testosterone, dihydrotestosterone and estradiol levels. Brains were removed for histological analysis.

### Behavioral tests

#### Open field test

The open field (OF) paradigm was originally introduced as a measure of emotional behavior, but it is also a suitable test for the evaluation of general motor activity in animal models [15]. The maze consisted of black wood square arena (60x60x30 cm). The rats were placed in the centre of the arena and spontaneous exploration activity was recorded during five minutes. The following parameters were scored: total distance moved (TDM), velocity, percentage of time moving, cumulative duration in the centre zone and frequency in the centre zone.

#### Elevated plus maze test

The elevated plus maze (EPM) test is used for the estimation of anxious-like behavior. EPM consisted of two opposite open (50x20 cm) and two opposite enclosed arms (50x20x30 cm), elevated 100 cm from the floor. Each rat was placed in the centre of the elevated plus maze facing the open arm, and was allowed 5 minutes for free exploration. The following parameters were estimated: the cumulative duration in open arms, the frequency in open arms, total distance moved (TDM), velocity, percentage of time moving, the number of rearings and the number of head-dippings. Those parameters are considered as indicators of anxiety level [39, 40]. In order to estimate the overall exploratory activity in EPM test, we introduced a new parameter that included both patterns of exploratory activity observed in the EPM test (the number of rearings and the number of head-dippings), since they are taking place in different zones of EPM (closed and open arms, respectively) in different time intervals, and presented it as total exploratory activity—TEA episodes (the sum of the numbers of rearings and head-dippings).

#### Evoked beam-walking test

Evoked beam-walking (EBW) test was performed in order to estimate emotional reactivity of animals by means of anxiety-provoking pattern effects on the performance in previously recorded beam-walking test [41]. Test was performed using apparatus consisted of white wooden box with the hole, as an escape box, for motivating the animal to cross the beam and the stainless steel beam (100x3x2 cm) fixed between the base of the escape box (100 cm above the floor) and a vertical stainless steel pole (60 cm above the floor). Rats were pre-trained to cross the beam (four trials were performed with 15 minutes intervals). At the start of the trial, the rat was placed at the end of the beam opposite to the escape box and the time to cross the beam was recorded. Fifteen minutes after the first recording, the rats were placed in the same starting position and the experimenter started tapping (approximately every second) with metal stick at the base of pole, while the rat traversed the beam (anxiety-provoking pattern), until the rat reached the escape box [42]. The results were expressed as percentage of shortening the time to cross the beam between trials.

#### Video recording system and analysis

OF and EPM tests were recorded by digital camera, mounted above mazes at the appropriate height. Video files were analyzed using Ethovision software [XT 10 base, Noldus Information Technology, the Netherlands].

### Serum hormone assays

Serum samples were assessed for total testosterone and estradiol levels by Elecsys 2010 analyzer using the method of electrochemiluminescence immunoassay (ECLIA). Standard commercial kits (Elecsys Testosterone II and Estradiol III, Roche Diagnostics, Mannheim, Germany) were used and the testosterone and estradiol levels were expressed in ng/ml and pg/ml, respectively. The sensitivities of the assays for testosterone and estradiol were 0.025 ng/ml and 5 pg/ml, respectively. Inter- and intra-assay coefficients of variance for testosterone and estradiol were 3.8 and 2.2, and 5 and 3.9%, respectively. Serum dihydrotestosterone was measured by sensitive kit (ALPCO Diagnostics, Salem, NH, USA) using ELISA method, and the values were expressed in pg/ml. The sensitivity of the assay for dihydrotestosterone was 6.0 pg/ml. Inter- and intra-assay coefficients of variance for dihydrotestosterone were 5.9 and 3.9%, respectively.

### Immunohistochemistry

Following decapitation, rat brains (after immediate removal from the skull) proceeded previously described procedure [23]—fixation in 4% neutral buffered formaldehyde, dehydration and were embedded. 5 μm thick coronal brain sections were dewaxed, rehydrated and treated with citrate buffer (pH 6.0) in the microwave for antigen retrieval. Endogenous peroxidase activity was blocked with 3% H_2_O_2_, and nonspecific labeling was blocked by a commercial protein block (Novocastra, UK). Slices were incubated in primary antibody—mouse monoclonal anti-PV (1:1000, Sigma-Aldrich) overnight at room temperature. Labeling was performed using biotin-conjugated secondary antibody, followed by streptavidin-HRP, and visualization was done with 3,3’-diaminobenzidine (DAB) chromogen (all components from Peroxidase Detection System RE 7120-K, Novocastra, UK). Finally, sections were counterstained with Mayer’s hematoxylin and covered. Counting was done on Leica DM4000 B LED microscope with digital camera Leica DFC295 using Leica Application Suite (LAS, v4.4.0) software system. Unilateral (alternately left or right hemisphere) assessments of the hippocampal PV-immunoreactive cells was performed for all animals (n = 40). The number of immunoreactive neurons was always obtained on the dorsal hippocampus (level of section was 3.8 mm caudal to the bregma, according to Paxinos and Watson stereotaxic atlas [43]) on one hippocampal section per animal, and expressed per 1 mm^2^ of the investigated hippocampal region (CA1, CA2/3 and dentate gyrus—DG). Two independent experimenters who made the counts were blind to the experimental protocols and showed high inter-rater reliability (Pearson's r = 0.95), and the mean value was taken as the final count.

### Statistical analysis

The data presented herein were expressed as the means ± S.E.M. Parameters obtained in behavioral tests were initially submitted to Levene's test for homogeneity of variance and to Shapiro-Wilk test of normality. Comparisons between groups were performed using One-way ANOVA, followed by Bonferroni post hoc analysis, for behavioral tests parameters and serum hormones levels, and with Scheffe’s post hoc test for morphological analysis. Pearson's coefficient of correlation was used to analyze relationships between parameters obtained in behavioral tests and histological data, and simple linear regression analyses were performed. A value of p<0.05 was considered to be significant. Statistical analysis was performed with SPSS version 20.0 statistical package (IBM SPSS Statistics 20).

## Results

### Behavioral tests

#### Open field test

**Fig 1:**
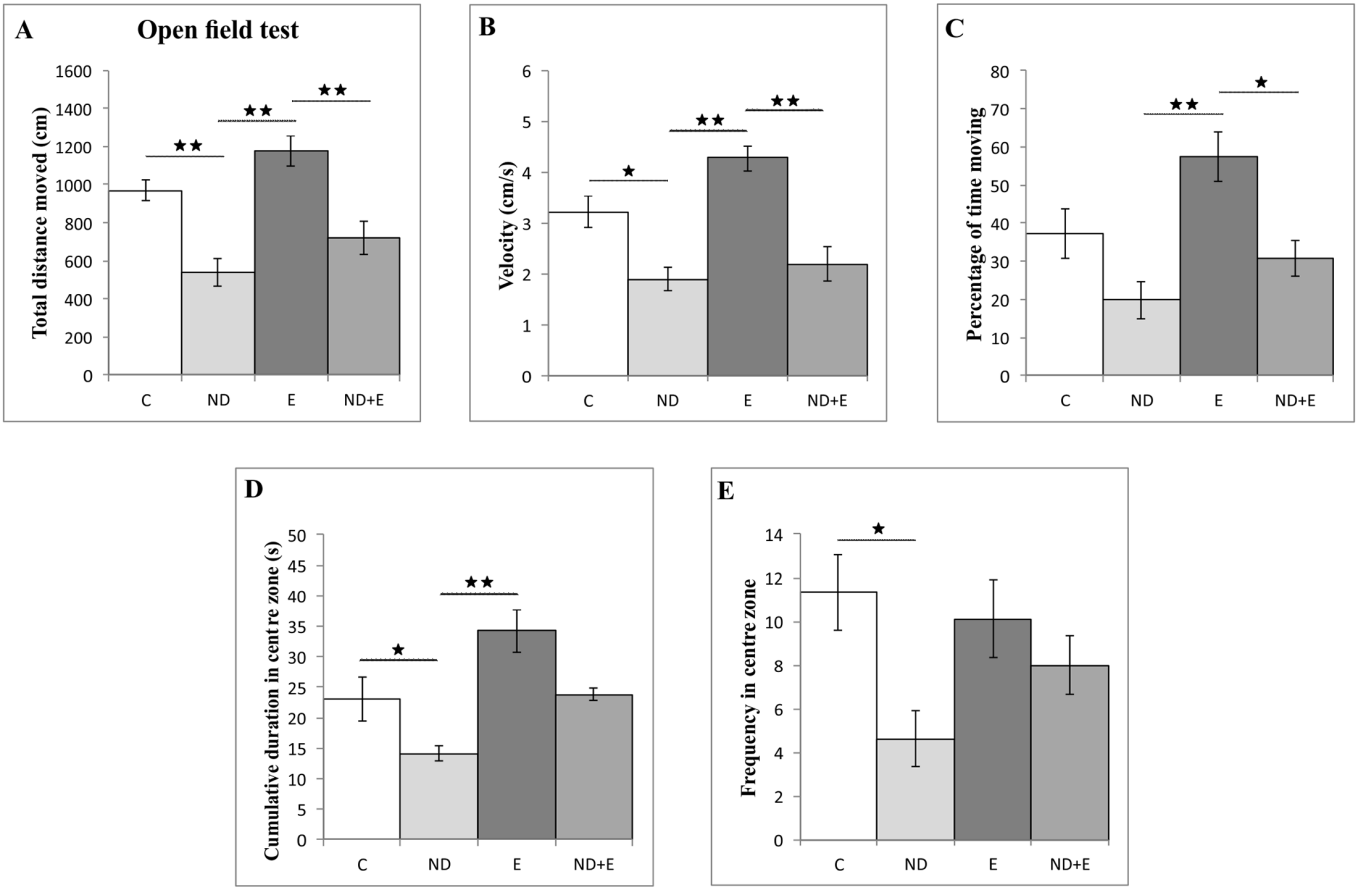
Parameters calculated from the open field test. C—control group, ND—nandrolone decanoate group, E—exercise group, ND+E—nandrolone decanoate plus exercise group. (Mean ± SEM, * denotes a significant difference p<0.05, ** denotes a significant difference p<0.01, n = 8 per group One-way ANOVA, Bonferroni post hoc analysis).

The parameters of locomotor activity—total distance moved (Fig 1A), velocity (Fig 1B) and percentage of time moving (Fig 1C), obtained in OF test (data in S1 Table, section A) were significantly reduced in the ND group compared to the exercise (F = 14.102, 14.416 and 7.712, respectively, df = 3, p<0.01) and control group (p<0.01 for TDM and p<0.05 for velocity). Chronic ND treatment along with exercise also significantly decreased locomotor activity by means of the same parameters compared to the exercise group (p<0.01 for TDM and velocity, and p<0.05 for percentage of time moving). ND significantly lowered cumulative duration (Fig 1D) and frequency in the centre zone (Fig 1E) compared to the control group (F = 9.581 and 3.601, respectively, df = 3, p<0.05). Although with no change compared to control, exercise significantly increased cumulative duration in the centre zone compared to ND group (p<0.01).

#### Elevated plus maze test

**Fig 2:**
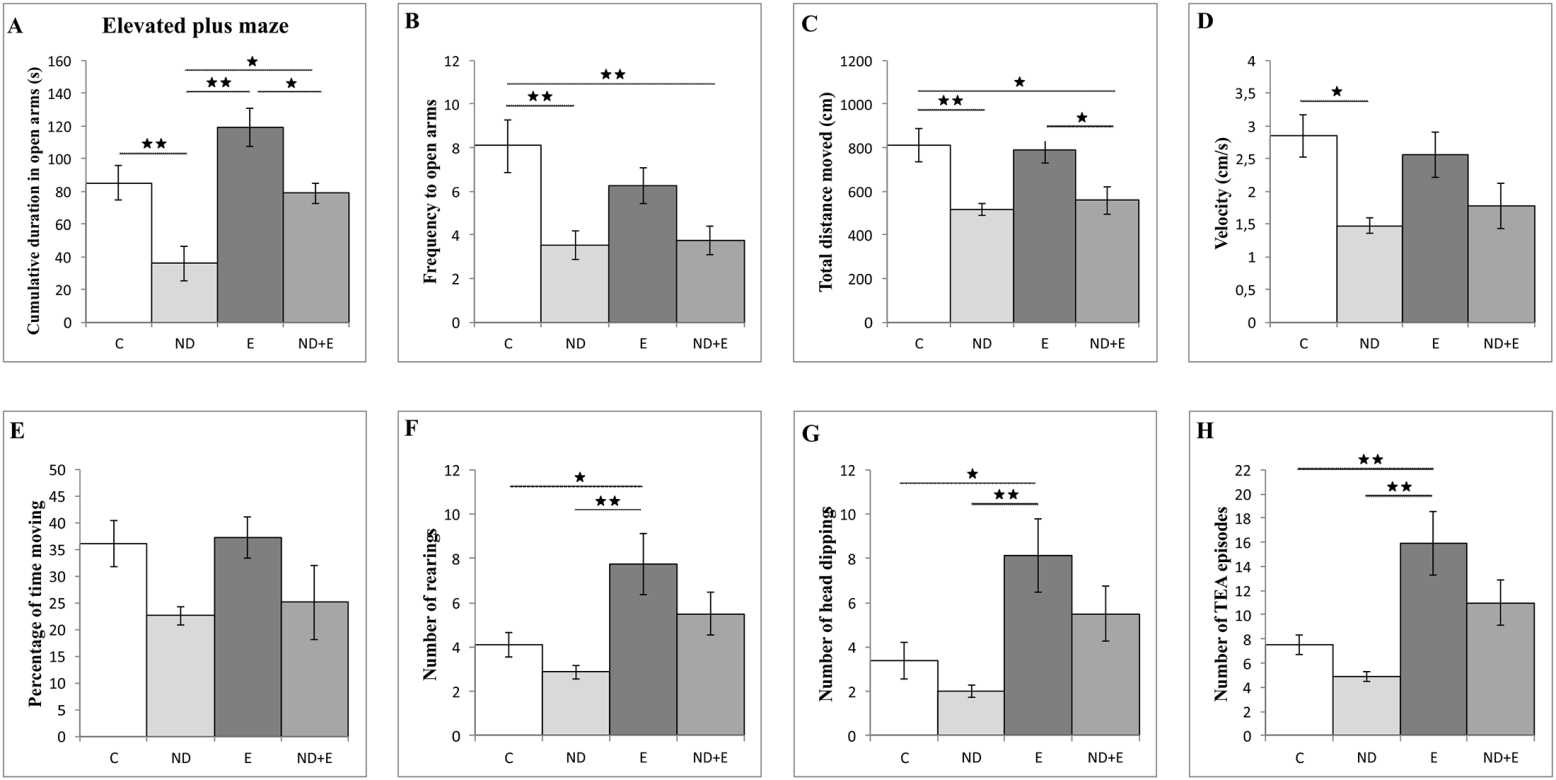
Parameters calculated from the elevated plus maze test. C—control group, ND—nandrolone decanoate group, E—exercise group, ND+E—nandrolone decanoate plus exercise group. (Mean ± SEM, * denotes significant difference p<0.05, ** denotes a significant difference p<0.01, n = 8 per group, One-way ANOVA, Bonferroni post hoc analysis).

ND significantly reduced the total time (Fig 2A) and the frequency in open arms (Fig 2B) compared to control (F = 11.976 and F = 6.291, respectively, df = 3, p<0.01). With no effect compared to control, exercise protocol alone induced significant increase in the cumulative duration in open arms compared to ND group (p<0.01). Results from the combined group (data in S1 Table, section B) showed that exercise also attenuated ND induced reduction in cumulative duration, while the frequency to open arms remained significantly lower compared to control group (p<0.01). The effect of exercise observed in combined group was sufficient to significantly increase in the cumulative duration in open arms compared to ND group (p<0.05). Locomotor activity observed in EPM was significantly reduced by chronic ND treatment when compared to the control group by means of reduction in TDM (Fig 2C) and velocity (Fig 2D) (F = 6.611 and 4.668, p<0.01 and p<0.05, respectively, df = 3). This effect of ND was obvious even in the combined group, with significant decrease in TDM compared to control (p<0.05). There was no significant alteration in the percentage of time moving following any of applied protocols. Exploratory activity in EPM, expressed by means of the number of rearings (Fig 2F), the head-dippings (Fig 2G) and TEA episodes (Fig 2H), in the exercise group was significantly (F = 8.179, 5.693 and 5.448, respectively, df = 3) increased compared to the control group (p<0.05 for the number of rearings and the head-dippings, p<0.01 for TEA). ND administration resulted in significant decrease in all three parameters for evaluation of exploratory activity in EPM test compared to exercise group (p<0.01). However, beneficial effect of exercise was also manifested in combined group by reversing the ND induced reduction of exploratory activity to the control values.

#### Evoked beam-walking test

**Fig 3:**
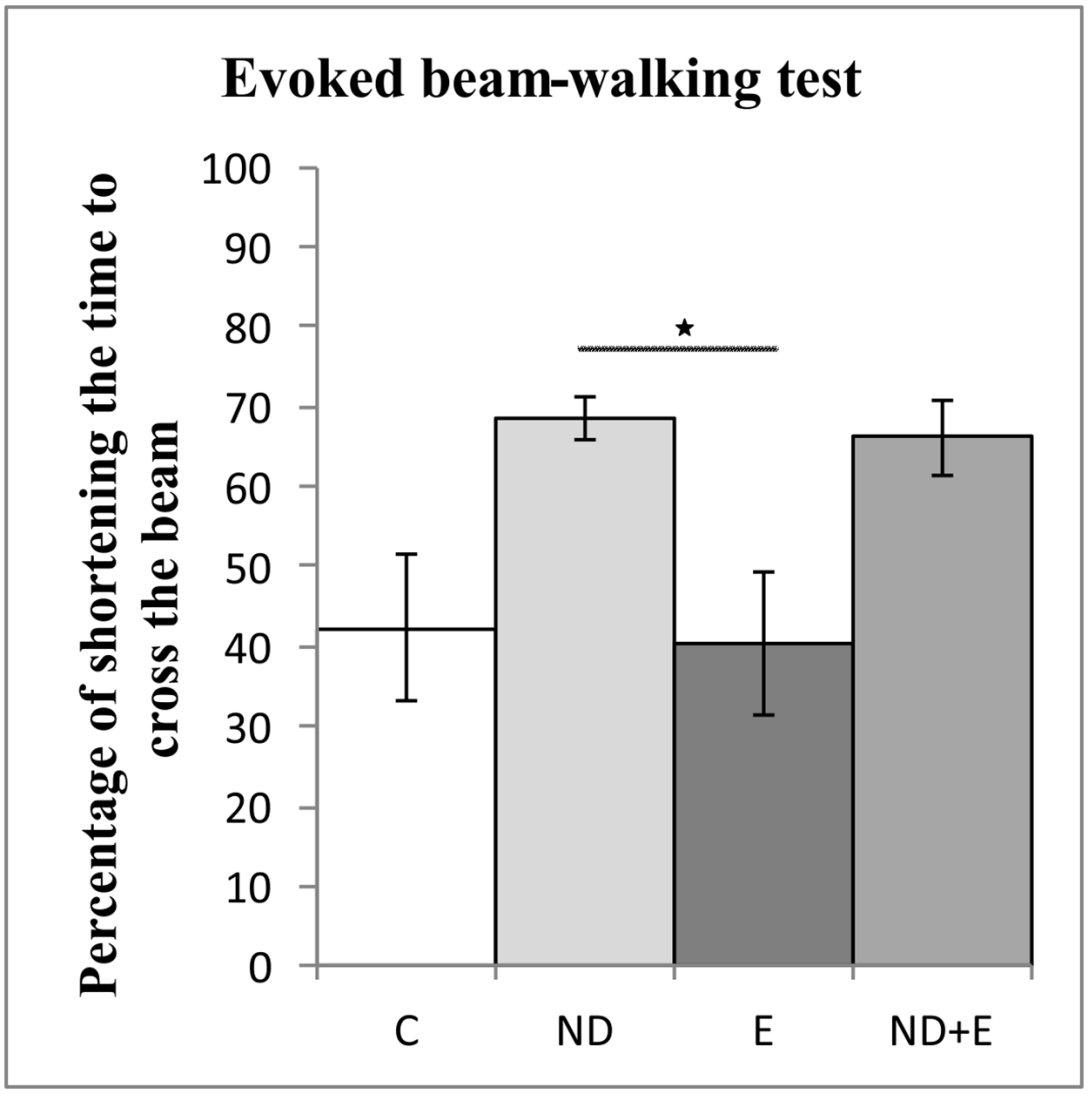
Parameters calculated from the evoked beam-walking test. C—control group, ND—nandrolone decanoate group, E—exercise group, ND+E—nandrolone decanoate plus exercise group. (Mean ± SEM, * denotes a significant difference p<0.05, n = 8 per group, One-way ANOVA, Bonferroni post hoc analysis).

As shown in Fig 3, neither exercise nor ND protocol induced significant change in the reduction of time to cross the beam compared to control (data in S1 Table, section C). However, chronic swimming training significantly enhanced the reduction of time to cross the beam, compared to ND group (F = 4.672, df = 3, p<0.05).

### Serum hormone levels

As shown in Table 1, both chronic ND administration and exercise induced significant increase in serum T level (F = 22.390, df = 3, p<0.01) compared to control group (data in S1 Table, section D). Simultaneous application of ND along with exercise protocol resulted in more prominent increase in T compared to control group (p<0.01). Serum DHT levels were not affected following ND application (Table 1). However, exercise protocol significantly increased DHT levels (F = 10.328, df = 3) compared to all other groups (p<0.01). Chronic ND administration significantly increased serum E2 levels (F = 13.228, df = 3) compared to control values (p<0.01). ND induced increase in serum E2 levels was also manifested in combined group, leading to significant increase in E2 compared to control (p<0.01) and exercise group (p<0.05).

**Table 1:** Serum levels of testosterone, dihydrotestosterone and estradiol.

Group	C	ND	E	ND+E
Hormone				
T (ng/ml)	1.57 ± 0.14	6.54 ± 0.54 ^a^**	5.48 ± 0.64 ^b^**	7.25 ± 0.65 ^c^**
DHT (pg/ml)	519.75 ± 86.83	641.12 ± 54.50	1051.12 ± 98.78 ^b^** ^d^**	554.12 ± 54.78 ^e^**
E2 (pg/ml)	13.10 ± 0.95	20.87 ± 0.90 ^a^**	18.08 ± 0.97	24.49 ± 2.07 ^c^** ^e^*

Values are expressed as mean ± SEM. C—control group, ND—nandrolone decanoate group, E—exercise group, ND+E—nandrolone decanoate plus exercise group.

^a^ C vs. ND;

^b^ C vs. E;

^c^ C vs. ND+E;

^d^ ND vs. E;

^e^ E vs. ND+E;

* denotes a significant difference p<0.05,

** denotes a significant difference p<0.01, n = 8 per group, One-way ANOVA, Bonferroni post hoc analysis).

### Immunohistochemistry

**Fig 4:**
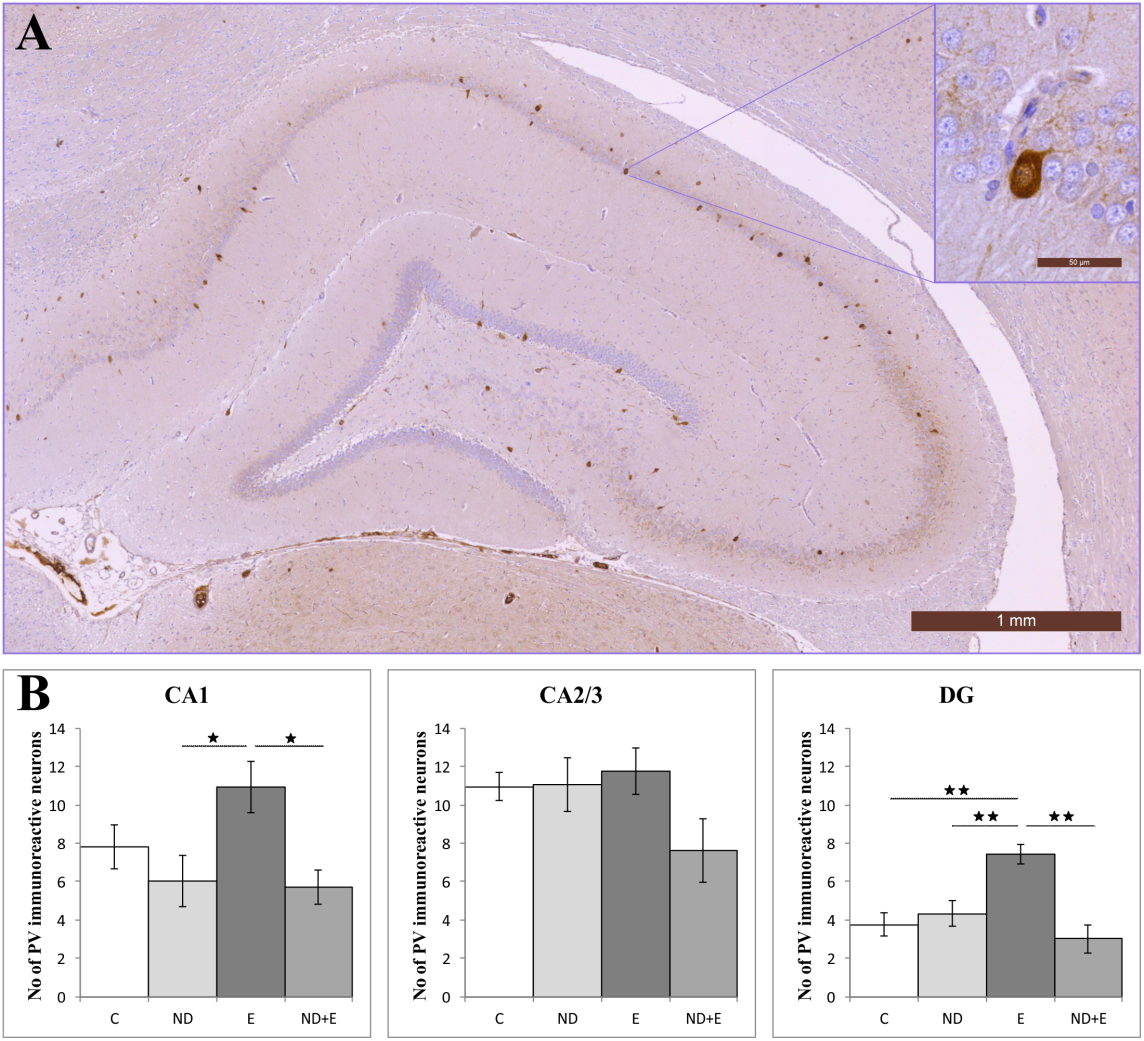
Immunohistochemical expression of PV positive interneurons in the rat hippocampus. (A) Distribution in the control group. (B) Number of PV immunoreactive neurons in hippocampal regions. C—control group (n = 8), ND—nandrolone decanoate group (n = 12), E—exercise group (n = 11), ND+E—nandrolone decanoate plus exercise group (n = 9). (Mean ± SEM, * denotes a significant difference p<0.05, ** denotes significant difference p<0.01, One-way ANOVA, Scheffe’s post hoc test).

Parvalbumin interneurons in all investigated groups (data in S1 Table, section E) were located mostly within or in vicinity of pyramidal cell layer in CA1 and CA2/3, and mostly in granular cell layer in DG (Fig 4A). The analysis of PV expression showed statistical significance between groups in two regions, CA1 and DG, of the hippocampus (F = 5.232 and F = 12.050, respectively, df = 3). As shown in Fig 4B, statistical analysis of PV immunoreactive neurons showed that neither exercise nor ND protocol induced significant change in the number of these cells in CA1 region compared to control group. However, chronic ND administration resulted in significant decrease in the number of PV immunoreactive neurons in CA1 compared to exercise group (p<0.05). The ND induced reduction in PV positive neurons was still preserved in combined group compared to exercise group (p<0.05). In the DG exercise significantly increased the number of PV immunoreactive neurons compared to control (p<0.01). Although ND treatment did not significantly influenced the number of PV interneurons in DG compared to control, their number was significantly lower compared to exercise group (p<0.01). Moreover, the reduction in number of PV interneurons in DG induced by ND administration was still obvious in combined group, maintaining the number of PV positive interneurons significantly below the values observed in exercise group (p<0.01).

**Fig 5:**
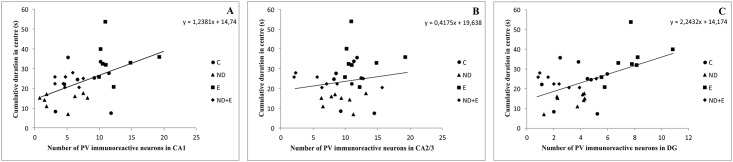
Relationship between the number of PV immunoreactive neurons in different regions of hippocampus (A—CA1, B—CA2/3, C—DG) and the cumulative duration in the centre zone observed in the OF test (n = 8 per group). Simple regression analysis indicated that the number of PV neurons in CA1 (F = 10.946, df = 1, Pearson's r = 0.52, p = 0.002) and DG (F = 12.715, df = 1, Pearson's r = 0.55, p = 0.001) was positively correlated with time spent in centre zone. There was no significant correlation for CA2/3 region (F = 0.674, df = 1, Pearson's r = 0.15, p = 0.42).

**Fig 6:**
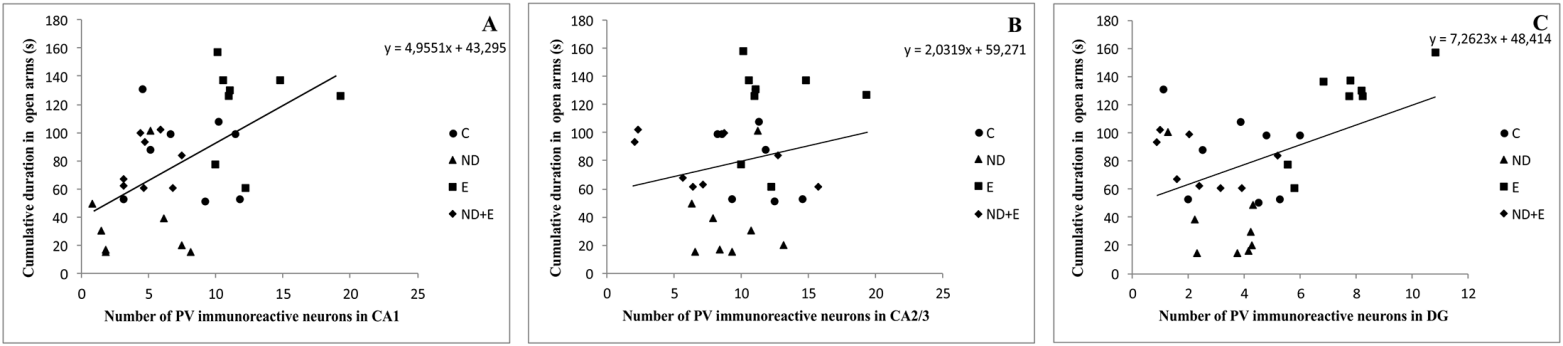
Relationship between the number of PV immunoreactive neurons in different regions of hippocampus (A—CA1, B—CA2/3, C—DG) and the cumulative duration in the open arms in the EPM test (n = 8 per group). Simple regression analysis indicated that the number of PV neurons in CA1 (F = 11.320, df = 1, Pearson's r = 0.52, p = 0.002) and DG (F = 7.481, df = 1, Pearson's r = 0.45, p = 0.01) was positively correlated with cumulative duration in open arms. There was no significant correlation for CA2/3 region (F = 1.032, df = 1, Pearson's r = 0.18, p = 0.31).

**Fig 7:**
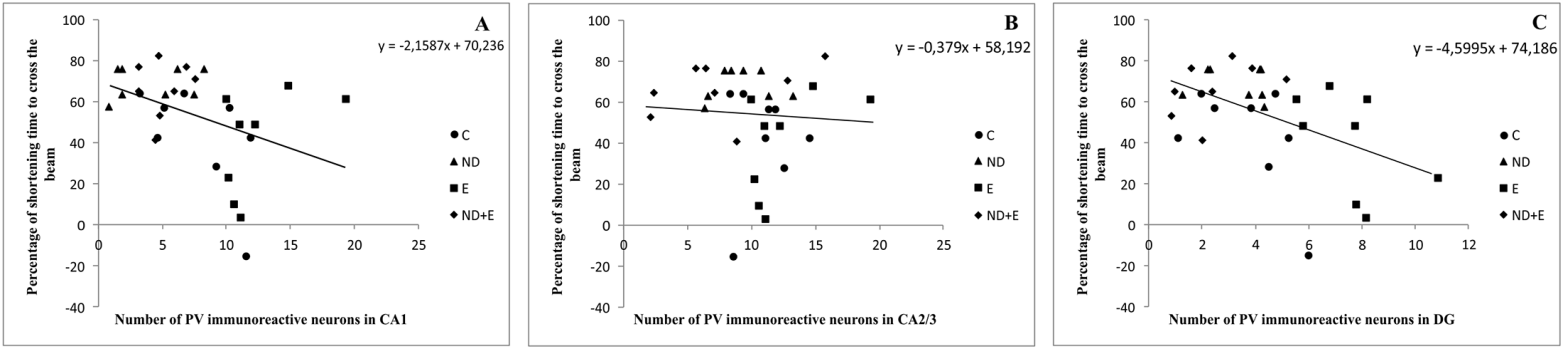
Relationship between the number of PV immunoreactive neurons in different regions of hippocampus (A—CA1, B—CA2/3, C—DG) and the shortening the time to cross the beam in the EBW test (n = 8 per group). Simple regression analysis indicated that the number of PV neurons in CA1 (F = 5.617, df = 1, Pearson's r = 0.40, p = 0.02) and DG (F = 9.621, df = 1, Pearson's r = 0.50, p = 0.004) was negatively correlated with the shortening of time to cross the beam. There was no significant correlation for CA2/3 region (F = 0.106, df = 1, Pearson's r = 0.06, p = 0.75).

As shown in Fig 5, simple regression analysis revealed that the number of PV immunoreactive neurons in CA1 and DG was significantly positively correlated with the cumulative time spent in the centre zone of the open field, with no significant correlation for the number of immunoreactive neurons in CA2/3 region. The number of PV neurons in CA1 and DG (Fig 6) was also significantly positively correlated with the cumulative duration in open arms in EPM test. On the other hand, the regression analysis showed that the number of PV neurons in CA1 and DG was significantly negatively correlated with the reduction of time to cross the beam in EBW test, with no significant correlation with the number of immunoreactive neurons in CA2/3 region (Fig 7).

## Discussion

The main findings in this study are that both chronic swimming training and chronic exposure to ND had a significant influence on behavioral patterns in rats. Also, all three applied protocols were sufficient to induce alterations in the sex hormone profile (Table 1). The data concerning the effects of AASs (including ND) on sex hormone levels are very controversial. It has been reported that prolonged ND treatment resulted in decrease of serum T in rats [44–46], and mice [47]. However, the elevation of serum T level observed in this study is in line with numerous reports that confirmed T levels increase following both acute [48] and chronic [49–52] AASs (ND and other testosterone derivatives) administration in rats. The elevation of T in rat serum, as observed in this study, could be explained by increase in T production due to stimulation of endogenous production in Leydig cells [53]. The other explanation for this level of T serum increase could be found in the possibility that either endogenous and exogenous testosterone (or testosterone like substances) were measured [49, 54]. Simultaneously, chronic exposure to AAS resulted in increase in E2 serum levels, as observed in our study, was also previously reported in rats [49, 55]. The elevation of E2 was not observed following stanozolol administration, due to lack of aromatization to estradiol [55]. However, the influences of AASs administration on DHT levels were rarely evaluated. It has been reported that although with the weak effect of ND alone, the stacking of different AASs caused significant elevation in DHT plasma level [49].

Like for the AASs, the data for the impact of exercise on sex hormone levels are also divergent. It has been reported that mild exercise did not affect T and DHT levels in plasma [28]. On the other hand, intensive swimming exercise produced decrease in T plasma levels [56]. However, the elevation of T, DHT and E2 (not significant) levels following the exercise protocol performed in this study is in accordance with previous reports that showed the increase in T [50], and E2 [57] after stepwise increasing exercise in rats. The reasonable explanation for different sex hormone levels could be found in marked variability of exercise protocols. However, even in the case exercise had no significant effect on sex hormone levels in blood, it has been reported that exercise altered sex hormone concentrations in hippocampus [28]. The elevation in T, DHT and E2 in hippocampus was accompanied with increased neurogenesis. The impact of sex hormone levels on neurogenesis was confirmed by means of three postulated mechanisms: increase in cell proliferation, differentiation and cell survival [28]. Those mechanisms do not necessarily include actions via androgen and estrogen receptors in hippocampus. On the other hand, it has been reported that ND action on neurogenesis in the rat brain produced completely opposite effect. ND administration severely affected (decreased) neurogenesis, as well as androgen receptor expression in hippocampus [58], that can lead to behavioral changes. However, colocalization of sex hormones receptors with specific hippocampal regions assigned for behavioral control (such as GABAergic system in CA1, CA2/3 and DG), that would allow better insight in sex hormone actions in modulation of behavior, still has to be evaluated.

The results obtained in OF test indicate that chronic exposure to ND produced anxiogenic effect (lowered TDM, percentage of time moving and the velocity) confirming previously described anxiogenic-like effect of AASs in OF test [59]. In contrast to the anxiogenic effects of the ND treatment, chronic swimming training induced typical anxiolytic behavior in rats when compared to anabolic-induced effects in OF test, although that effect was not significant compared to control. Therefore, when compared to ND, exercise increased TDM, velocity (the parameters that are used as indicators of anxiety-related behavior [60, 61]) and the percentage of time moving, suggesting anxiolytic effect of chronic swimming training. Since there is no evidence for the effects of swimming on those specific parameters obtained in OF test, we can only compare them to the data obtained with different exercise protocols. Our results are similar with the report considering chronic wheel running and treadmill protocols [6], but they also can be compared to the previously described variable anxiolytic effect of exercise by means of similar parameters of OF test in rats [62]. Possible explanation for those contradictory results may be found in different exercise protocols. Exercise also increased the cumulative duration in the centre zone compared to anabolic-treated animals (the increase was not significant compared to control group), but not the frequency to the centre zone of OF. Both parameters are commonly used as reliable indicators of anxiety level in OF [63]. The anxiolytic effects of exercise, confirmed by alteration of those two specific parameters of motor behavior in the centre zone of OF, was previously described in rats [13]. Combined effects of chronic exposure to ND and swimming protocol resemble the combination of two opposite individual effects. Observed effect of exercise, by means of parameters of OF test, was abolished when this protocol was combined with ND to the level that was below the control values, suggesting that the effects of ND overcome the beneficial influence of exercise, as it was reported for testosterone undecanoate [15].

Anxiolytic effect of chronic swimming training (compared to both ND treated and control groups) was also observed in EPM test, by means of increase of the cumulative duration in open arms of the EPM, that are along with the number of rearings and head-dippings considered as common indicators for behavioral alterations of anxiety origin [39]. Although observed anxiolytic-like effect of chronic exercise is not in accordance with previously described running (treadmill and wheel) protocols [6], it correlates with reported anxiolytic effects of swimming protocol in mice [64] and wheel running in rats [13]. Those differences may be explained by the lack of standardization of performed protocols (various types of exercise (forced or voluntary), differences in duration and load), that can differently affect brain and behavior [65]. The chronic exposure to ND induced the opposite effect on anxiety level parameters in the EPM test. Our results for the ND effects in EPM do not correlate with reported anxiolytic-like effect of high dose of ND [66], but they are in line with described anxiogenic-like effects of ND [7] and other AAS in rats [67]. Reported differences in AAS effects on anxiety levels in the EPM test may appear due to different AAS doses and protocols used in those trials. Also, ND abolished exercise-induced increase of the cumulative duration in open arms, as well as frequency to open arms. Beside, the results for exploratory activity in EPM test observed in this study confirmed the benefit of a new parameter, the number of TEA episodes, since it allowed to determine anxiolytic effect of exercise on exploratory activity in EPM (more significant increase in the number of TEA episodes), comparing to two commonly used parameters (the number of rearings and head-dippings–Fig 3).

EBW test results confirmed anxiety-related behavioral changes. The exercise protocol did not significantly affect the time to cross the beam indicating that overall anxiolytic-like effect of exercise (markedly expressed in the previous test) had minor effect in this task. On the other hand, chronic exposure to supraphysiological dose of ND decreased the time to cross the beam in EBW test compared to exercise group.

Results of our study indicate the opposite effects of long-term exercise and supraphysiological doses of AAS on the number of PV immunoreactive neurons in different regions of hippocampus. Those adverse effects were the most obvious in CA1 and DG, with no difference in CA2/3. Namely, exercise protocol performed in this study increased number of PV immunoreactive neurons by almost 40%, while AAS chronic administration decreased PV neurons by 25% in CA1 region (not significant). The effect of simultaneous administration of ND along with swimming protocol observed in the combined group on hippocampal PV interneurons was almost the same as in the AAS group, suggesting the dominant effect of testosterone derivatives on hippocampal plasticity under the protocol performed in our study, as previously described in rat hippocampal CA1 region [68]. Alteration in hippocampal structure by means of the number of PV immunoreactive neurons was also noticed in DG, where exercise almost doubled (90%) number of PV neurons, with no significant change in number of PV neurons followed AAS administration alone, as well as in combination with an exercise protocol. Our results are in line with previous reports concerning the effects of different exercise protocols on the increase of PV positive cell number in DG, with no change in CA1/CA3 [24]. On the other hand, different training protocols resulted in increase of number of PV immunoreactive neurons in CA1 and CA2/3, while number of PV interneurons in DG was unaffected [25, 26]. However, the effects of chronic AAS administration on hippocampal plasticity by means of the number of PV immunoreactive neurons have not been reported yet.

Observed anxiolytic effect of exercise by means of parameters of behavioral tests in this study was accompanied with the increase of PV interneurons in hippocampus. Beneficial effect of exercise on hippocampal GABAergic system has been attributed to specific cell proliferation and neurogenesis [24]. Still, it remains unclear whether the increase in PV immunoreactivity occurs in exercise-induced neurogenesis [69] or in previously formed cells, suggesting complexity of hippocampal response to exercise.

The impact of hippocampal function on anxiety has been analyzed in numerous studies [70]. The results for the correlation between the number of hippocampal PV neurons and behavioral patterns that could be considered as indicators of increased anxiety levels observed in this study, revealed significant connection between those histological and functional changes. Since the major alterations in hippocampal plasticity were observed in CA1, it seems that this immunohistochemical change may be causally connected to anxiogenic effect of ND. Furthermore, indirect evidence for such functional-morphological interplay may be found in attenuation of beneficial effects of exercise by ND (evidenced in both behavioral and histological analysis), that was even more pronounced than the effect of ND itself.

In summary, presented results provide a confirmation of beneficial influence of exercise by means of clear anxiolytic effects observed in the battery of tests designed for the estimation of anxiety. Exercise-induced behavioral alterations were accompanied with a significant increase in the number of PV interneurons in hippocampus. On the other hand, chronic treatment with ND in the dose sufficient to mimic AAS abuse in humans induced mild decrease in hippocampal PV neurons, followed by anxiety-like behavioral changes. Also, applied supraphysiological dose of ND was sufficient to attenuate beneficial effects of exercise in rats by means of decreased exercise-induced anxiolytic effect, as well as to reverse exercise-induced augmentation in the number of immunoreactive PV neurons in the hippocampus. Those results implicate the possibility that alterations in the hippocampal PV interneurons (i.e. GABAergic system) may be involved in the modulation of anxiety levels induced by AAS abuse and/or extended exercise protocols.

## Supporting information

S1 Table Raw data for the parameters obtained in this study. (XLSX)

## Abbreviations

AAS: anabolic androgenic steroid
DHT: dihydrotestosterone
E2: estradiol
EBW: evoked beam-walking
EPM: elevated plus maze
ND: nandrolone decanoate
OF: open field
PV: parvalbumin
T: testosterone
TDM: total distance moved
TEA: total exploratory activity
